# Management of Spontaneous Corneal Perforation in Undiagnosed Primary Sjögren’s Syndrome

**DOI:** 10.7759/cureus.82440

**Published:** 2025-04-17

**Authors:** Amine Razzak, Hala Ait Ammar, Loubna Mouhib, Mohamed Bouazza, Mohamed Elbelhadji

**Affiliations:** 1 Ophthalmology, Mohammed VI University of Sciences and Health, Casablanca, MAR

**Keywords:** conjunctival flap, corneal perforation, dry eye, keratitis, primary sjögren's syndrome

## Abstract

Spontaneous corneal perforation is a true diagnostic and therapeutic emergency that can reveal autoimmune diseases such as primary Gougerot-Sjögren syndrome (pSS). We report the case of a 59-year-old female patient with no notable medical history who presented with a painful unilateral red eye and decreased visual acuity. Examination revealed a unilateral paracentral corneal perforation, which was subsequently confirmed to be associated with pSS based on the positivity of anti-SSA/SSB antibodies and a minor salivary gland biopsy showing lymphocytic infiltration, without any signs suggestive of an associated systemic disease. The patient underwent conjunctival covering and received systemic treatment with corticosteroids and immunosuppressants, leading to a favorable clinical outcome. This case highlights the importance of early diagnosis and a multidisciplinary approach to prevent severe complications associated with pSS.

## Introduction

Primary Sjögren’s syndrome (pSS) is a chronic systemic autoimmune disorder characterized by lymphocytic infiltration of exocrine glands, particularly the salivary and lacrimal glands. It is one of the most common autoimmune diseases after rheumatoid arthritis [1]. Among its most frequent and severe manifestations is ocular dryness due to lacrimal hyposecretion. Severe dryness can lead to corneal complications ranging from superficial punctate keratitis (SPK) to corneal ulcers, threatening visual prognosis [2]. Corneal perforation secondary to Sjögren Syndrome, though rare, is a serious complication requiring urgent diagnosis and management.

We report a case of corneal perforation as the initial presentation of pSS. Medical-surgical management stabilized the disease and preserved satisfactory visual acuity.

## Case presentation

A 59-year-old woman with no significant medical history, including no history of toxic habits or previous herpetic keratitis, presented to the emergency department with painful red eyes and decreased visual acuity. She reported chronic ocular discomfort, including a foreign body sensation, and xerostomia.

**Figure 1:**
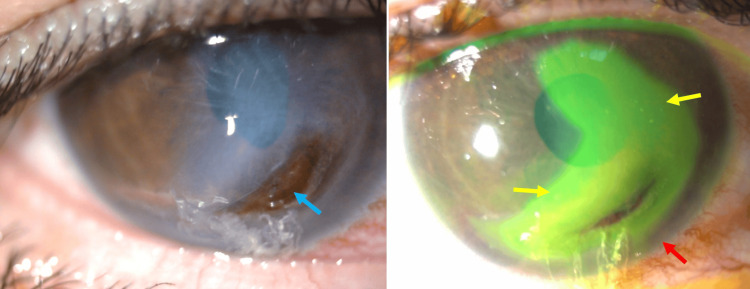
Preoperative appearance of the right eye Left: Inferior perforation without inflammatory infiltrates (blue arrow). Right: Extensive superficial ulcer (yellow arrow), separated from the limbus by healthy cornea (red arrow).

Initial examination revealed a visual acuity of 1/20 in the right eye and 4/10 in the left eye. Biomicroscopy of the right eye showed an infero-paracentral corneal perforation (four to seven o’clock), sparing the central cornea. It was surrounded by a large fluorescein-positive corneal ulcer with a clean base and was separated from the limbus by healthy corneal tissue. The anterior chamber was flat (Figure 1).

**Figure 2:**
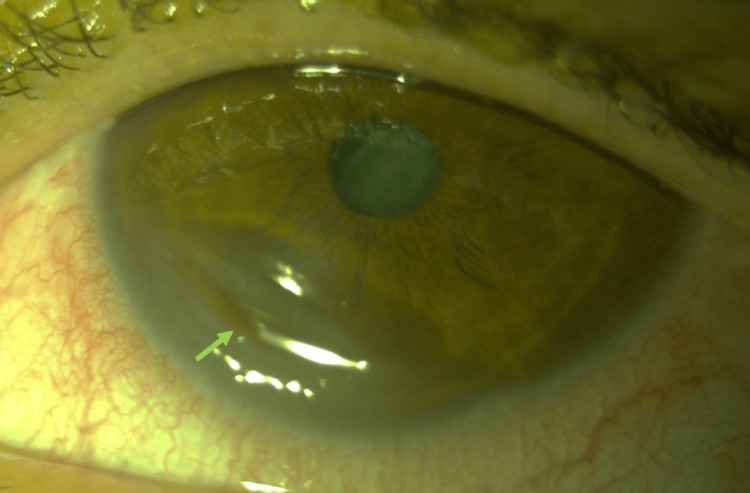
Preoperative appearance of the left eye Inferior stromal thinning (green arrow).

The left eye exhibited an inferonasal crescent-shaped paracentral ulcer (six to eight o’clock) with a clean base, stromal thinning without white infiltrates, and dense SPK (Figure 2).

Schirmer’s test confirmed severe aqueous tear deficiency (<5 mm in five minutes bilaterally), and the tear breakup time (TBUT) was less than 5 seconds in both eyes. Corneal sensitivity was preserved. The patient was urgently hospitalized. Due to the unavailability of an amniotic membrane, a partial sliding conjunctival flap was performed on the right eye. Medical treatment included preservative-free lubricants, autologous serum eye drops (ASED), topical cyclosporine, oral doxycycline, intravenous methylprednisolone bolus (followed by oral prednisone at 1 mg/kg/day, tapered gradually), and azathioprine.

**Figure 3:**
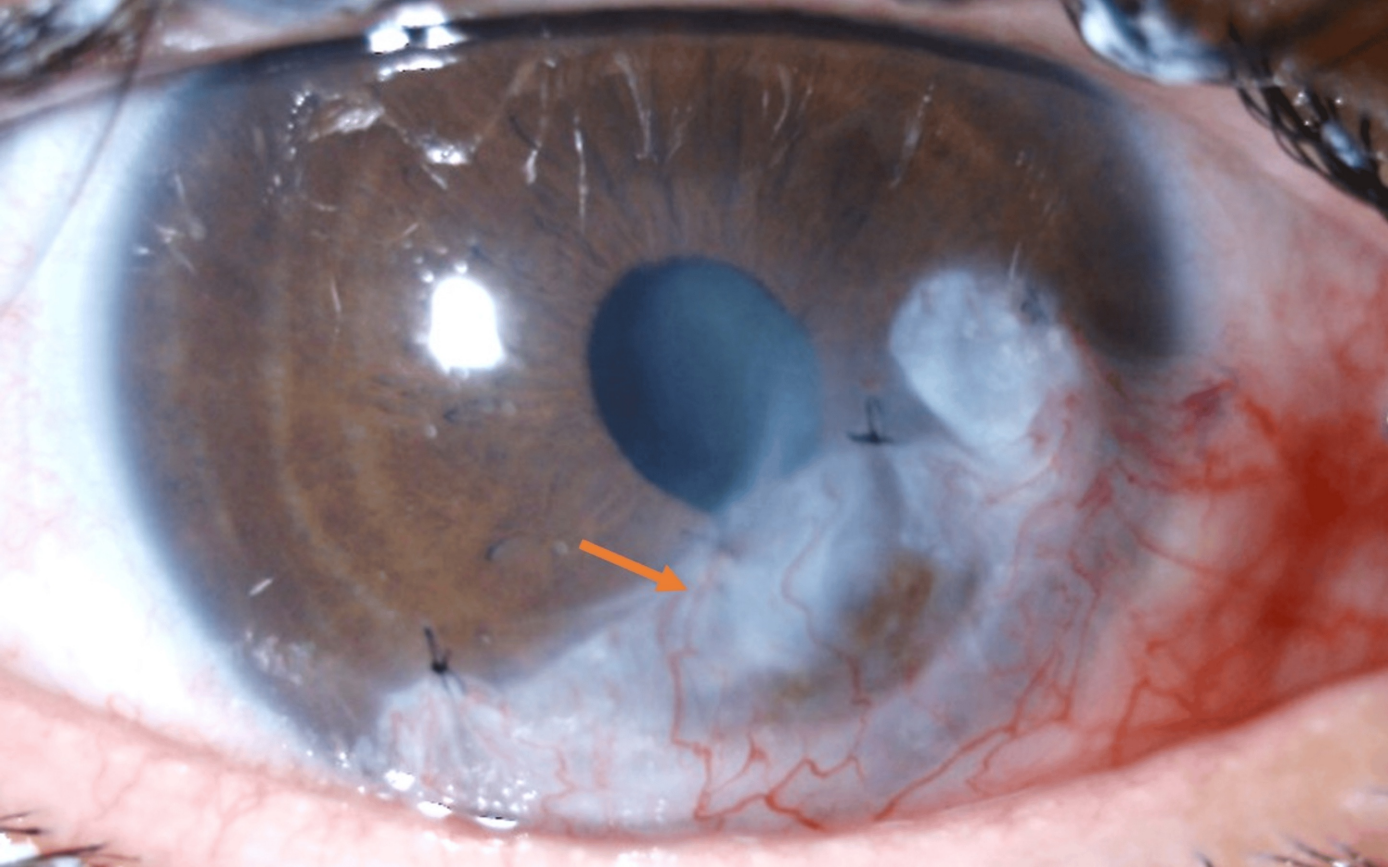
Postoperative appearance of the right eye on day seven Restored anterior chamber after partial inferior conjunctival flap (orange arrows).

Clinical evolution was favorable, with anterior chamber reformation in the right eye, improved visual acuity bilaterally (4/10 in the right eye and 7/10 in the left), and complete corneal ulcer healing. However, peripheral corneal thinning persisted in the right eye (Figure 3).

Etiological workup revealed positive anti-SSA/SSB antibodies. Minor salivary gland biopsy confirmed lymphocytic infiltration. No signs of associated systemic disease were found, confirming primary SS.

**Figure 4:**
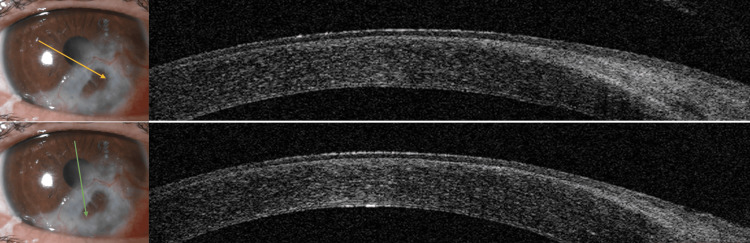
AS-OCT images performed at 12 months postoperatively The arrows indicate the orientation of the OCT scan.

At 12-month follow-up, the patient remained stable with no recurrence or new complications. There were no symblepharon, fornix shortening, or endothelial dysfunction. Corrected visual acuity remained stable at 4/10 despite the presence of a residual corneal opacity sparing the visual axis, localized inferior iridocorneal adhesion, and moderate astigmatism corrected with glasses. Figure 4 shows the AS-OCT image obtained in 12 months.

**Figure 5:**
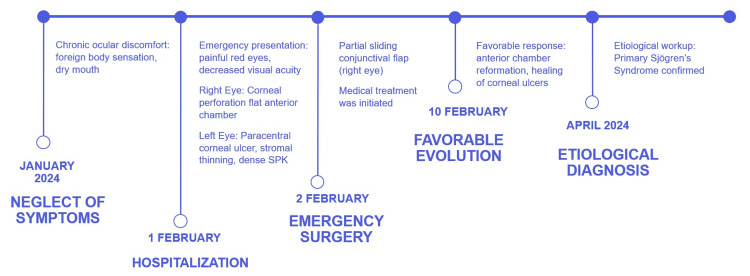
Timeline of the clinical case from the first clinical symptoms to the etiological diagnosis

A corneal graft was proposed as a secondary surgical treatment, but the patient declined, considering her visual acuity satisfactory. Figure 5 summarizes the clinical timeline.

## Discussion

Sjögren’s syndrome is a chronic systemic autoimmune disease marked by lymphocytic infiltration of exocrine glands, presenting as dry eye, xerostomia, and arthritis. Its prevalence ranges from 0.1% to 0.8%, with a female predominance [3]. SS may be primary (as in our case) or secondary to systemic diseases like rheumatoid arthritis [4].

Diagnosis relies on clinical, serological, radiological, and histological criteria. However, dry eye, the hallmark ocular manifestation, is often underdiagnosed due to its multifactorial nature [5]. Severe dry eye in SS can lead to complications such as SPK or, rarely, corneal perforation, an ophthalmologic emergency requiring multidisciplinary care [6,7]. In our case, the diagnosis of pSS was established due to the absence of clinical or paraclinical signs suggestive of a systemic disease. Moreover, the absence of a history of stimulant abuse allowed us to rule out toxic keratopathy.

Risk factors for perforation include ocular surgery, nonsteroidal anti-inflammatory drugs (NSAID) eye drops, diagnostic delays, or poor treatment adherence [8]. In our case, delayed consultation and diagnosis due to the patient's neglect did not allow for early therapeutic management to prevent spontaneous perforation. Early diagnosis of dry eye syndrome, based on tear film assessment and ocular surface analysis, would have allowed for timely and effective management, reducing the risk of severe complications.

The differential diagnoses of corneal melting include Mooren’s ulcer, peripheral ulcerative keratitis (PUK), and corneal involvement in systemic autoimmune diseases such as rheumatoid arthritis, granulomatosis with polyangiitis, or systemic lupus erythematosus [7]. PUK is often associated with systemic autoimmune conditions and is characterized by crescent-shaped peripheral stromal thinning, epithelial defects, and frequently adjacent scleral inflammation [4]. In the absence of an identified systemic disease, Mooren’s ulcer, a diagnosis of exclusion, must be considered. It usually presents as a painful, progressive peripheral ulceration starting at the limbus with an initial inflammatory infiltration that evolves into an ulcer spreading circumferentially, often with marked inflammation [4]. In our patient, the absence of peripheral stromal infiltration and scleral involvement, combined with positive anti-SSA/SSB serological markers and a minor salivary gland biopsy confirming the diagnosis of primary Sjögren’s syndrome without evidence of associated systemic disease, allowed us to rule out these differential diagnoses. Additionally, the clinical presentation with a paracentral ulcer associated with healthy corneal tissue, without peripheral white infiltrates, was not suggestive of PUK or infectious keratitis; therefore, microbiological cultures were not requested. The paraclinical workup also did not reveal any evidence of another systemic disease.

Management of corneal perforation involves surgical closure, ocular integrity preservation, and inflammation control. Therapeutic options depend on perforation size, location, and ocular surface status. Soft contact lenses aid pre-perforative ulcers [2]. Small perforations may be treated with cyanoacrylate glue, multilayered amniotic membrane grafts, or patch grafts [9]. Larger perforations require penetrating or lamellar keratoplasty [6], though graft availability may limit these options. These techniques can also be combined with conjunctival excision, especially in cases of progressive marginal ulceration, to reduce inflammatory mediators. In our case, given a confirmed peripheral corneal perforation with an area of healthy cornea separating it from the limbus, we opted for a partial conjunctival flap.

The choice of a partial conjunctival flap was based on both anatomical considerations and practical constraints. The corneal perforation was paracentral and peripheral, which made it suitable for flap coverage without compromising the visual axis. While cyanoacrylate glue is often a good option for small corneal perforations, it was not appropriate in this case due to the size of the leak and the irregular margins. Amniotic membrane transplantation and lamellar and crescent keratoplasty are well-established techniques for managing such cases; however, they were not feasible due to the lack of tissue availability and the urgent need for surgical intervention. Given these limitations, the conjunctival flap provided a readily available, rapid, and effective solution for tectonic support and ocular surface protection. This low-cost technique achieved satisfactory results by closing the defect and preserving vision. This approach can be relevant in low-resource settings where the amniotic membrane or corneal graft is currently unavailable.

Inflammation control is critical. Intravenous corticosteroids followed by oral tapering stabilize acute inflammation. Preservative-free lubricants, autologous serum, and topical cyclosporine improve ocular surface health [10]. Systemic immunosuppressants like azathioprine reduce steroid dependence, while oral tetracyclines inhibit collagenase [4,11].

Multidisciplinary collaboration between ophthalmologists, internists, and rheumatologists is essential. Patient education on treatment adherence and avoiding self-medication is crucial to prevent complications.

## Conclusions

Corneal perforation is a rare but severe complication of primary Sjögren’s syndrome that can lead to significant visual impairment if not promptly diagnosed and treated. This case highlights the importance of recognizing ocular manifestations as a possible initial sign of an underlying autoimmune disorder. Severe dry eyes, often underdiagnosed, can progress to corneal ulceration and perforation, requiring urgent intervention. A multidisciplinary approach involving ophthalmologists, rheumatologists, and internists is crucial for effective disease management. A combination of surgical intervention and systemic therapy is crucial for stabilizing the disease and preserving vision. Long-term follow-up is necessary to monitor for recurrence and prevent further complications.

Early diagnosis, patient education, and adherence to treatment play a key role in preventing severe outcomes. Greater awareness among healthcare providers about the ocular complications of Sjögren’s syndrome is crucial for reducing diagnostic delays and improving patient outcomes.
